# New Disease

**Published:** 1864-04

**Authors:** 


### NEW DISEASE.

The addition of a new disease to the already enormous catalogue of the ills to which flesh is heir, can hardly prove a source of congratulation, the more especially if it happened to be one of the most loathsome which has yet been made known or conceived. But in calling attention to the newly-recognised "trichina disease," we have at least the satisfaction of knowing that we have here not a malady absolutely new, but only possessing features of novelty in so far as by the recognition of its singular and painful cause we have made a great step towards ensuring its prevention; for this is one of the diseases which is to be averted rather than cured.

The trichina disease is a febrile disorder depending upon the lodgment and migrations in the human body of multitudes of a microscopic worm (Trichina spiralis), which find their way into the economy through the eating of pork infested with the parasite, and pass in crowds from the intestines to the muscles, where they become encapsuled.

The history of this worm, its migration, and the disease it causes, is a marvelous chapter, yet very imperfectly known, in vital philosophy. The disease is one of which the reality and the serious importance cannot be contested. Müller, a German physician sends us in an original paper, which we publish in another column, a most careful observation of the symptoms of the disease, watched by him in a recent *epidemic* of the trichina fever in Hettstaedt, a small town in Prussia containing about 6000 inhabitants, which commenced in the middle of October last, in consequence of the infected persons having eaten a kind of sausage (not thoroughly cooked), made of pork in which were trichinæ. Some account of this epidemic has already appeared in several of the public journals, and it has necessarily created a profound impression: for in this small population eighteen to twenty persons had died from the disease, and more than eighty persons were at one period afflicted with the same malady, produced by the same cause.

The evidence of the nature of the disease is of the most positive and irresistible kind. In all the cases examined, the worm is found by microscopic examination in immence numbers in the muscles inspected, whether the examination be made by *harpooning* small portions of muscle during life—when a piece of living muscle the size of a hemp-seed has been found to contain no less than seven trichinæ—or by the *post mortem* examination of the muscles, which discovers everywere numbers of these pernicious worms. The symptoms of the disease are well marked. In this most recent outbreak at Hettstaedt, they are thus described by our contributor, Dr. Müller:—

"According to the information I obtained on the spot, the disease begins, a few days after eating the meat in which there were trichinæ, with loss of appetite, and almost without exception with diarrhœa and fever; œdema of the eyelids; also pain, or at least painful sensation of weakness in the limbs; œdema of the joints; difficulty in moving the tongue; profuse clammy prespiration; and those patients who do not become convalescent, die either unconscious with symptoms of typhus fever, or in a few cases, remain conscious to the end, complaining of inability to breathe freely.

"The only important symptom of typhus absent in the disease is the enlargement of the spleen, and it is very probable that some of the so-called epidemics of typhus fever in former days were caused by the propagation of trichinæ in the human body."

Similar symptoms were observed and described in an outbreak at Planen in Saxony, where from twenty-five to thirty persons were affected; and at Madgeburg, where the disease is said to have prevailed during five summers, but at first not to have been recognised. A symptom on which other observers than Dr. Müller have laid much stress, but to which he does not allude, is excessive and singular muscular pain, generally through the body, but especially in the calves of the legs, which become hard and swollen. This was so noticeable at Madgeburg that the disease was called Scleroma adultorum. In a very remarkable case recorded by Dr. Friedreich, of Heidelberg, where there were these excessive muscular pains, and the calves of the legs are described as being "hard and elastic, with a feel almost of india-rubber," the disease was diagnosed during life. The patient, who was treated with picro-nitrate of potash, slowly recovered.

"The muscles (calves of the legs) were harpooned several times. The first time was about twenty or twenty-one days after the commencement of the attack: although a piece of muscle only about the size of a hemp-seed was taken out, on less than seven trichinæ were found. Ten days later other harpooning showed no trichinæ; but seven days after, a living, but not encapsuled, trichina was found; and four days after this an encapsuled worm was discovered. Seventeen days later, when the patient was quite well, the search for trichinæ was fruitless. The muscular fibres were not inflamed, but were fattily degenerating rapidly. A very extraordinary discovery was that a trichina was found in the pus of one of the boils, so that Friedreich asserts that the furunculoid disease was caused also by a wandering of the worm beyond its usual site. The patient had been engaged in killing pigs the week before his illness, and had often held his bloody knife in his mouth, and had also eaten raw some of the bits intended for sausages."

We might be well satisfied to take comfort from the observation that these terrible records are from German sources, and that the sausage-eating propensities of the German may perhaps explain how they suffer from a parasitic disease non-existent here. There are, however, some important facts bearing upon that view of the case which cannot be safely disregarded. In the first place, the Trichina spiralis was originally discovered in the human body in 1835, by a distinguished English observer (Professor Owen) in a specimen of human muscle submitted to him by Mr. Paget, then a very young man. Hilton and Wormald had previously noticed a speckled condition of the muscles in some subjects, and it was this that Owen made out in 1835 to be due to "white specks in the muscles, and seem to be cysts of an elliptical figure, with the extremities in a general attenuated, elongated, or more opaque than the body (or intermediate part) of the cyst, which is in general sufficiently transparent to show that it contains a minute coiled-up worm." Professor Owen gives, in the paper communicated to the Zoological Society of London, from which this extract is taken, a very complete description of this worm, to which, however, Henle, Virchow, Leuckart, Luschka, and Kuchenmeister have added further details. They have especially traced its life-history, and their researches show that "the fully-developed trichina is a distinct filiform worm, occupying the alimentary canal, and giving birth to young trichinæ, which pierce the walls of the intestines, and on reaching the muscles become capsulated."

The evidence that the Trichina spiralis is bred in man by his feeding on pork infested with this parasite is of the most positive and direct character. Professor Gamgee, in his recent article on Diseased Pork and Microscopic Parasites in man, in the *Popular Science Review*, gives a useful picture of the symptoms of the disease in animals, to which all may refer with advantage; for it is by recognising the disease with care in animals which are materials for human food that danger to man will be best avoided. He dwells with force and propriety on the necessity of scrutinizing the habits of the domestic pig. He points out that parasitic maladies in the pig specially abound in Ireland, where swine live most amongst human beings; and draws inferences of practical importance to feeders and to all who eat pork.

It is a remarkable fact that we get from eating pork the two other most destructive parasites which prey upon the human body—the tapeworm and the hydatid. Leuckart has shown, and it is now acknowledged, that trichinæ are not killed by salting or freezing pork, nor by its becoming putrid. Whether smoking kills them is not settled, but the imperfect smoking to which a great part of the preparations of pork are subjected certainly has not sufficed to destroy them. The observations of Wormald, Hilton, Paget, Bellingham, and others show that this disease—not looked for, because almost unknown to us during life as a disease—certainly exists amongst us. It resembles so much "continued fever" in its simple characters that it may well be passed over by those who are not on the watch; but the recent observations of epidemics to which we have alluded, and the paper which we publish to-day from the pen of Dr. Müller, will put the profession on the alert; and it is to be earnestly hoped that, if it exist anywhere, it may now be immedately discovered, and the most rigid measures enforced of sanitary prevention.—*London Lancet.*

